# Young Mice Exhibit Increased Relative Ventricular Remodeling After AMI—A Longitudinal CMR Study

**DOI:** 10.1111/acel.70748

**Published:** 2026-09-30

**Authors:** Heiko Lemcke, Paul Wörner, Antonia Dalmer, Ralf Gäbel, Praveen Vasudevan, Matti Raitza, Moritz Schweins, Tobias Lindner, Hüseyin Ince, Bernd Joachim Krause, Brigitte Vollmar, Robert David, Felix G. Meinel, Cajetan Immanuel Lang

**Affiliations:** ^1^ Department of Cardiac Surgery Rostock University Medical Center Rostock Germany; ^2^ Department of Life, Light and Matter University of Rostock Rostock Germany; ^3^ Institute of Diagnostic and Interventional Radiology Rostock University Medical Center Rostock Germany; ^4^ Rudolf‐Zenker‐Institute for Experimental Surgery Rostock University Medical Center Rostock Germany; ^5^ Core Facility Multimodal Small Animal Imaging University Medical Center Rostock Germany; ^6^ Department of Cardiology Rostock University Medical Center Rostock Germany; ^7^ Department of Nuclear Medicine Rostock University Medical Center Rostock Germany; ^8^ Department for Preventive Sports Medicine and Sports Cardiology Technical University of Munich, School of Medicine and Health, TUM University Hospital Munich Germany

## Abstract

Cardiovascular diseases are the leading cause of mortality worldwide, with myocardial infarction (MI) contributing significantly to this burden. While age‐related differences in cardiac remodeling and healing post MI are well‐documented clinically, appropriate pre‐clinical studies are needed to evaluate the underlying pathological mechanisms and to further improve therapeutic interventions. Here, we compared LV remodeling between young and aged C57BL/6 mice, following permanent LAD ligation, using CMR imaging after 2, 7, and 28 days of MI induction. At baseline conditions, aged mice exhibited higher end‐diastolic and end‐systolic volumes and tended to have a reduced deformation capacity. Post‐MI, both groups showed significant LV dilation, loss of contractility, and impaired ejection fraction. Interestingly, young mice undergo larger proportional geometric changes relative to their pre‐infarction state. At the same time, histological data of labeled heart sections pointed to a more rapid healing process in young animals. These findings highlight the critical role of age in modulating cardiac remodeling and, thus, challenge the commonly held hypothesis of an age‐dependent increase of LV remodeling.

## Graphical abstract

Serial CMR demonstrated no age‐related aggravation of post‐MI remodeling, although old mice displayed larger baseline cardiac geometry and increased diastolic mass.

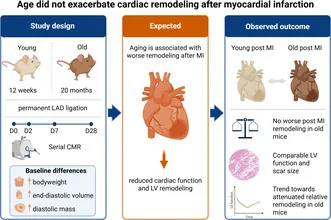

## 1 Introduction

Ischemic heart disease is the leading cause of death worldwide (Vaduganathan et al. 2022). Particularly, timely reperfusion of the occluded vessel has significantly reduced mortality of acute myocardial infarctions and the loss of functional myocardium (Fröhlich et al. 2013). Yet, a certain extent of myocardial necrosis is often inevitable and may lead to adverse left ventricular remodeling and eventually result in congestive heart failure. Despite huge advances in pharmacological therapies, mortality of heart failure remains comparable to some of the most common types of cancer (Mamas et al. 2017).

The challenge to replace damaged myocardium gave rise to the research field of myocardial regeneration (Olson 2024). In this context, mouse models have unique properties for investigating biological and molecular mechanisms of novel therapies and represent the most important model organism in this field (Patten and Hall‐Porter 2009).

The majority of MI patients are older than 60 years (Salari et al. 2023). In contrast, most pre‐clinical studies use mice aged around 12 weeks, when the animals have barely reached sexual maturity, likely corresponding to a human teenager (Dutta and Sengupta 2016) and thus limiting clinical translation to human MI patients.

Yet, age has been reported to significantly affect myocardial healing. The few existing studies have described an age‐dependent impairment of myocardial healing and more severe left ventricular remodeling and decline of contractile function (Bujak et al. 2008; Yabluchanskiy et al. 2015; Gould et al. 2002). Connective tissue homeostasis seems to be impaired with delayed granulation tissue formation and reduced collagen deposition (Bujak et al. 2008). Collagen Type V has recently been identified to play a crucial role in scar maturation around 7 days post MI (Yokota et al. 2020) and may be expressed at lower levels in aged mice.

Despite the importance of age in cardiac remodeling, there is a lack of comprehensive data comparing LV remodeling between young and aged animals, and longitudinal imaging studies are particularly scarce. Therefore, the aim of this study was the comparison of LV remodeling following permanent left anterior descending artery (LAD) ligation in young (12–13 weeks) and aged mice (20–21 months) using serial cardiac magnetic resonance imaging (CMR). Based on previous studies, we hypothesized that aged mice would exhibit significantly more pronounced LV remodeling with a larger scar and more dramatic decline in systolic function.

## 2 Methods

### 2.1 Study Design and Animal Model

In this prospective experimental study, we used a total of 21 young (12–13 weeks) and 17 old (20–21 months) female C57BL/6 mice. Animals were purchased from Janvier Labs. Mice were housed under a 12‐h light/dark cycle with unrestricted access to water and food. All procedures were conducted in accordance with relevant regulations, and the study protocols were approved by the local authority *Landesamt für Landwirtschaft, Lebensmittelsicherheit und Fischerei* Mecklenburg‐Vorpommern, LALLF, Germany (approval number 7221.3–1‐018/21). The ARRIVE guidelines were adhered to throughout the study.

### 2.2 LAD Ligation Procedure

The left anterior descending artery (LAD) ligation was performed by a senior researcher, highly experienced in murine myocardial infarction (MI) surgeries (Schweins et al. 2024). Initially, mice were anesthetized via intraperitoneal injection of pentobarbital sodium (50 mg/kg body weight) and subcutaneous administration of fentanyl (0.2 mg/kg). Following intubation with a 16‐gauge peripheral venous catheter (B. Braun), mice were ventilated using a small animal ventilator (MiniVent Type 845, Hugo Sachs Elektronik, Germany). Thoracotomy was performed using a surgical microscope (OPM 241 F, Carl Zeiss, Germany) and the LAD was ligated with an 8–0 prolene suture. Successful ligation was confirmed by the loss of color in the affected myocardial region. The thoracotomy wound was closed with DS19 Safil sutures.

Successful infarction was further confirmed by early LVEF assessment at Day 2 post MI, and animals with insufficient LVEF reduction were excluded from further analysis.

### 2.3 Cardiac Magnetic Resonance Imaging (CMR)

CMR scans were conducted 5 days prior and 2, 7, and 28 days after the LAD ligation (Figure 1). During the imaging sessions, mice were anesthetized with 1.5%–2.5% isoflurane in oxygen, and their respiration and body temperature were monitored using a physiological monitoring system (SA Instruments, Stony Brook, NY, USA). Imaging was carried out using a 7 Tesla small animal MRI system (BioSpec 70/30, Bruker BioSpin GmbH, Ettlingen, Germany) with a maximum gradient strength of 440 mT/m. A 112/86 mm volume resonator (transmit mode) and a 2 × 2 surface array coil (receive mode) were used, with mice positioned in a supine posture.

**FIGURE 1:**
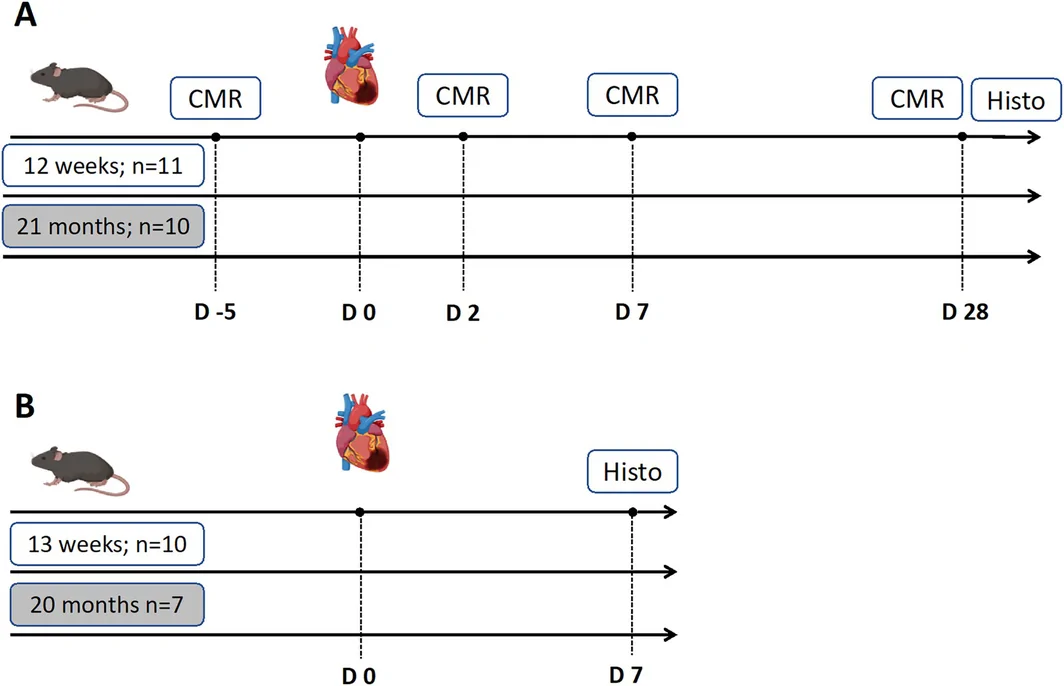
Study design. (A) 21 mice underwent longitudinal CMR imaging before LAD‐ligation as well as 2, 7, and 28 days after MI; hearts were excised for histology after the final scan. (B) Histology only was performed in a separate group of 17 animals.

### 2.4 The CMR Sequences Included

Planning scans were performed first. A short‐axis stack of 7 independent slices from the heart base to the apex, utilizing an integrated Cine‐FLASH sequence with navigator echo (Bruker), TE/TR: 2.2/8.6 ms, FA: 15°, FoV: 29.5 × 25.1 mm, matrix: 210 × 180, resolution: 140 × 140 μm, slice thickness: 1 mm, oversampling: 140%, 14 movie frames per slice, total acquisition time per slice: 2:46 min. Three long‐axis cine scans: 1‐, 2‐, and 3‐chamber views (same scan parameters as for the short‐axis stacks).

CMR data were analyzed using the software cvi42 Version 5.14.2 (2976) (Circle Cardiovascular Imaging, Calgary, Alberta, Canada). The epicardial borders were delineated using a semi‐automated tool, with corrections made by two experienced imaging experts. Functional parameters, including end‐diastolic volume (EDV), end‐systolic volume (ESV), stroke volume (SV), and left ventricular ejection fraction (LVEF), were determined. Diastolic mass was estimated from the short axis cine stack by contouring the endocardial and epicardial borders at end‐diastole. Myocardial volume was calculated as the difference between epicardial and endocardial volume across the slice stack and automatically converted by the software into diastolic mass using the standard myocardial density assumption. Strain analysis was performed to assess radial and circumferential strain in the short‐axis view (GRS_SAX_ and GCS) and radial and longitudinal strain in the long‐axis view (GRS_LAX_ and GLS_LAX_).

### 2.5 Histology

After cervical dislocation, the heart was removed, washed in 0.9% NaCl solution and embedded in air‐bubble‐free Tissue‐Tek O.C.T. Compound (Sakura Finetek, Alphen aan den Rijn, The Netherlands) on top of a brass stamp with the tip of the heart facing upwards. Embedded hearts were frozen in liquid nitrogen and stored at −80°C.

For the preparation of histological slides, frozen hearts were clamped in a microtome (microtome Kryostat CM1900 from Leica). In four planes (480 μm from the apex and between each plane) multiple 6 μm thin slices were cut and transferred onto microscope glass slides. Tissue slices were stained using the “Masson's Trichrome Stain Kit” (Polyscience) according to the manufacturer's instructions. Labeled heart slices were visualized with an Axiovert 40 CFL optical microscope (from Carl Zeiss) in 1 day with identical acquisition settings.

For quantitative scar analysis, the fibrotic area of stained heart sections was measured by computerized planimetry using Axio Vision LE Rel. 4.5 software (Carl Zeiss). Scar size was then expressed as the proportion of fibrotic area relative to the total heart area.

For antibody staining, slices were incubated with anti‐collagen V antibodies (Abcam, ab7046; polyclonal, rabbit IgG antibodies against cow, human or mouse collagen V), followed by treatment with goat anti‐rabbit IgG antibodies conjugated with Alexa Fluor 488 (Abcam, ab150077; polyclonal, goat IgG antibodies against rabbit IgG) as well as a DAPI. For visualization, slides were imaged using a confocal laser scanning microscope (ELYRA PS.1 LSM 780 fluorescence microscope from Carl Zeiss). Acquisition settings were kept the same between all recorded images. Images were taken as z‐Stack, followed by creation of a maximum intensity projection.

### 2.6 Statistical Analysis

Data are presented as mean ± standard error of mean. To assess normal distribution of datasets, the Shapiro–Wilk test was applied. In case of normally distributed parameters, we employed Welch's *t*‐test, while the Mann–Whitney test was used for nonparametric comparison of two groups. Longitudinal datasets for group comparison were analyzed in R using linear mixed‐effects models with age, time, and age‐by‐time interaction as fixed effects and animal ID as a random intercept to account for repeated measurements within the same animal. Baseline‐normalized data were analyzed analogously. For baseline‐adjusted analyses of absolute values, post‐MI time points (D2, D7, and D28) were modeled with the corresponding baseline value (D0) included as covariate. Fixed effects were tested using Type III ANOVA with Satterthwaite's approximation. Post hoc comparisons were based on estimated marginal means with Sidak or Dunnett adjustment as appropriate. Statistical significance was defined as *p ≤ 0.05, **p ≤ 0.01, ***p ≤ 0.001 and ****p ≤ 0.0001. Data visualization was performed using GraphPadPrism8 software (GraphPad Prism Inc., San Diego, CA, USA).

## 3 Results

### 3.1 LV Function and Morphology at Baseline Conditions

Initially, we compared baseline parameters for cardiac performance as well as ventricle volume and mass 5 days before LAD‐ligation for both groups (Figure 2).

**FIGURE 2:**
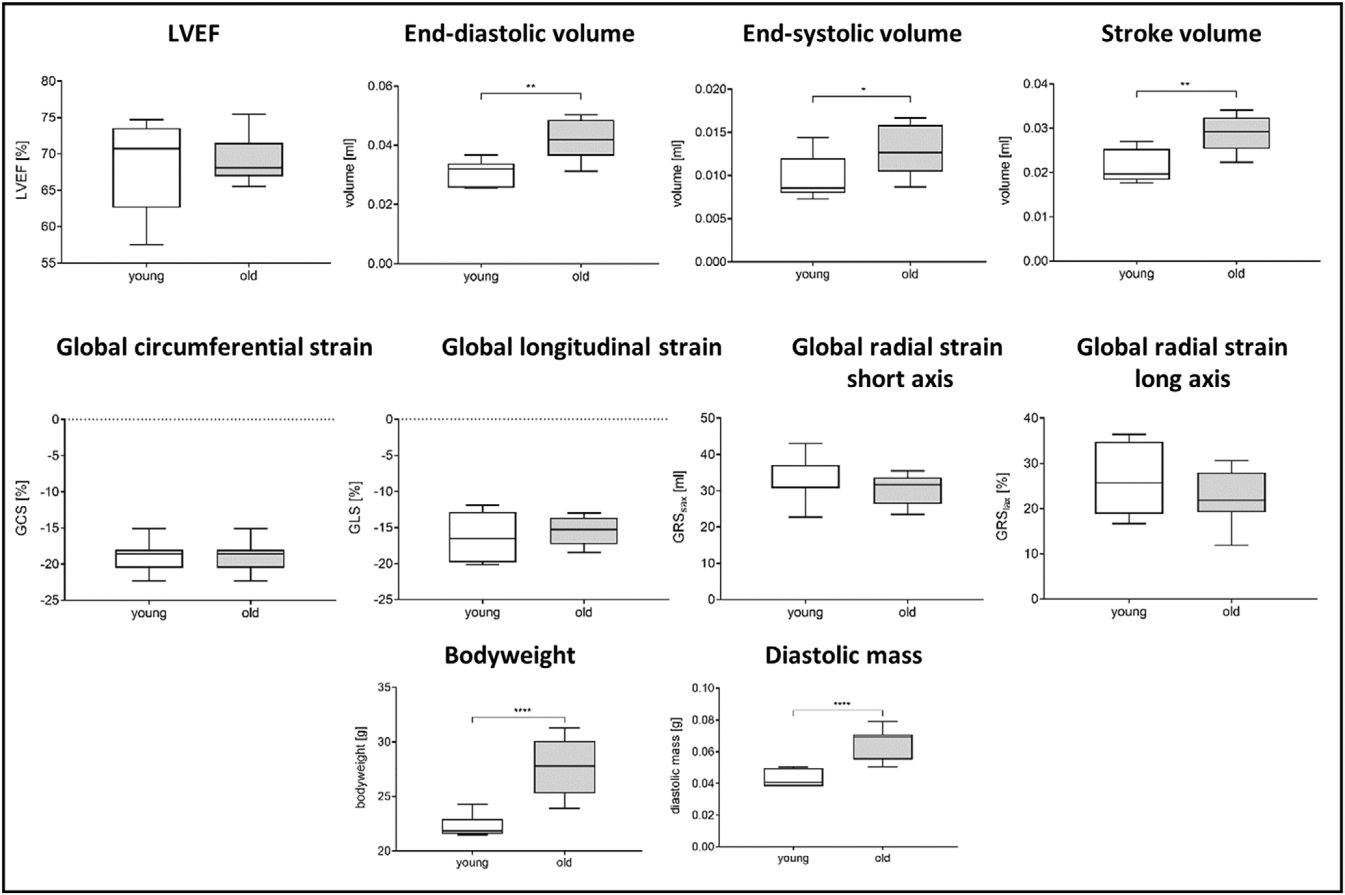
Baseline parameters for cardiac performance and heart contractility. Data were acquired from young and old animals 5 days prior to MI‐induction. While ventricle volume differs significantly, no age‐related changes were detected for ejection fraction and heart contractility. Welch's *t*‐test, *p < 0.05, **p < 0.01, ****p < 0.001, *n* = 7–9.

While ejection fraction (LVEF) was found to be similar in young and aged animals, old mice demonstrated an increased end‐diastolic and end‐systolic volume at baseline conditions. In accordance, the calculated stroke volume was significantly lower in young mice. Moreover, we evaluated the capacity of cardiac deformation by strain analysis of acquired CMR datasets. The quantitative analysis revealed elevated levels for all strain variants in young mice, but no statistical difference was found (Figure 2).

Bodyweight was found to be significantly higher in old than in young mice (Figure 2). To assess whether these differences in cardiac geometry and mass were attributable to body size, we fitted baseline linear models including age and body weight as covariates. For baseline EDV, age and body weight showed substantial overlap, and neither retained an independent effect when assessed conditionally in the same model (age: *p* = 0.366; body weight: *p* = 0.371). In contrast, for diastolic mass, body weight remained significantly associated with the parameter (*p* = 0.022), whereas age no longer showed an independent effect (*p* = 0.491).

### 3.2 LV Function and Morphology Following Permanent LAD Occlusion

In a next step, we investigated the differences of LV remodeling between young and aged mice after induction of MI. Mice were subjected to permanent LAD occlusion, and CMR imaging was performed after two, seven, and 28 days. As expected, we observed a marked decrease of LVEF in both groups 2 days after LAD occlusion that persisted until Day 28 (Figure 3A,B). Notably, young mice demonstrated a constant decline of LVEF over time, while aged mice showed a more robust LVEF from Day 2 after MI.

**FIGURE 3:**
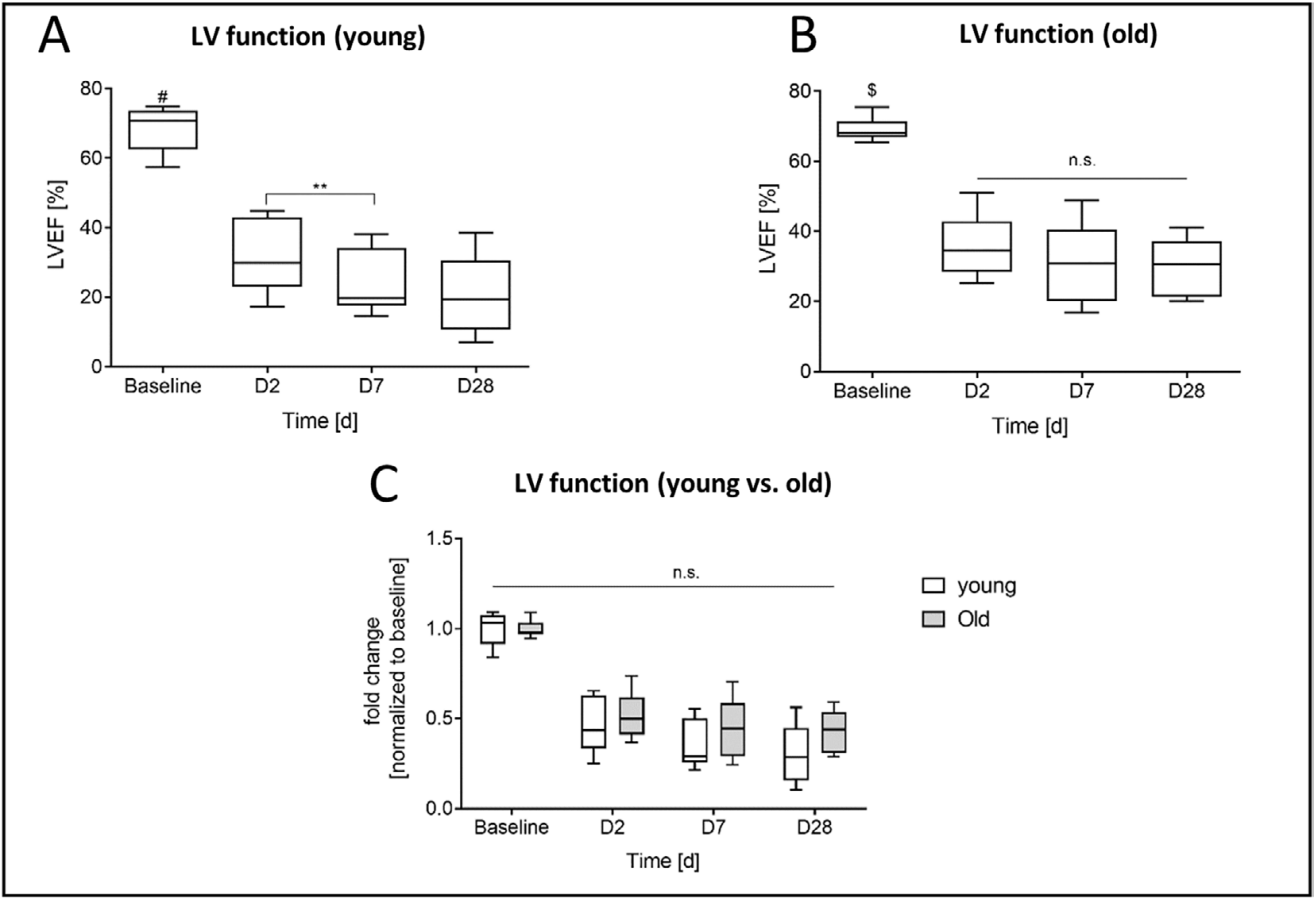
Impact of myocardial infarction on LVEF. (A) Young animals demonstrated a significant decline after LAD‐ligation (Baseline vs. D2; D2 vs. D7) that persists until D28. (B) In contrast, LVEF in old animals remains constant after the pronounced drop at D2. One‐way ANOVA with Sidak'ss post hoc test, #*p* < 0.001 Baseline versus D2, *p* < 0.0001 Baseline versus D7 and D28; $*p* < 0.0001 Baseline versus D2, D7, D28; **p < 0.005 (C) Comparison of baseline‐normalized data revealed no significant differences between both groups at any time point. Linear mixed‐effects model, three‐way ANOVA, followed by Sidak post hoc test, *n* = 7–11.

Comparison of absolute LVEF values demonstrated no significant difference in longitudinal post‐MI trajectories between young and old mice (Figure S1A). Consistently, baseline‐normalized LVEF values showed a significant effect of time, but no significant effect of age and no significant age‐by‐time interaction (Figure 3C), indicating similar relative impairment of LV function in both groups.

Besides the reduction in LVEF, LAD ligation induced marked left ventricular dilation in both young and old mice. Analysis of absolute end‐diastolic volume (EDV) and end‐systolic volume (ESV) demonstrated a pronounced increase in ventricular chamber size after MI, whereas stroke volume (SV) decreased (Table 1). Statistical analysis of the absolute values revealed strong time effects after MI (EDV: *p* = 2.19e‐13; ESV: *p* = 1.996e‐14; SV: *p* = 3.903e‐06). Although pairwise comparisons indicated significant differences between groups at selected time points, including Day 7 and Day 28 (Table 1), no significant age‐by‐time interactions were detected, indicating no robust difference in absolute longitudinal remodeling trajectories between young and old mice. Consistent with this, baseline‐adjusted follow‐up models did not reveal robust age effects at post‐infarct time points.

**TABLE 1:** MI‐induced changes ventricle volume in young and old mice.

	End‐diastolic volume [μL]		End‐systolic volume [μL]		Stroke volume [μL]	
	Young	Old	Young	Old	Young	Old
Baseline	30.8 ± 4.4	41.7 ± 6.6	9.7 ± 2.5	12.9 ± 2.8	21.0 ± 3.5	28.8 ± 4.1
D2	49.2 ± 8.1	52.5 ± 9.2	34.3 ± 8.7	33.9 ± 8.3	14.8 ± 3.4	18.5 ± 4.3
D7	79.3 ± 24.5	69.2 ± 14.9***	62.0 ± 23.3	48.8 ± 17.1**	17.2 ± 3.8	20.4 ± 4.9
D28	98.2 ± 34.2	84.9 ± 26.2***	80.6 ± 35.4	61.3 ± 24.8***	17.5 ± 5.6	23.5 ± 4.3

*Note:* Young animals demonstrated a smaller ventricle and stroke volume at baseline conditions. MI leads to a profound increase of ventricle volume in both groups. Students *t*‐test, **p < 0.005, *** *p* < 0.001, *n* = 7–11.

Because young mice started from substantially smaller baseline ventricular volumes (Figure 2), EDV and ESV were additionally normalized to each animal's pre‐infarction baseline. In these analyses, both EDV and ESV showed significant effects of age (EDV: *p* = 0.0042; ESV: *p* = 0.0077), time (EDV: *p* = 2.19e‐13; ESV: *p* = 1.996e‐14), and age‐by‐time interaction (EDV: *p* = 0.0035; ESV, p = *p* = 0.0064) (Figure 4A,B). Moreover, significant differences between groups were observed on Day 7 and Day 28 for both parameters. In contrast, baseline‐normalized SV showed a significant effect of time (*p* = 3.903e‐06), but no significant effect of age or age‐by‐time interaction (Figure 4C). Thus, while the absolute longitudinal trajectories did not differ significantly between groups, baseline‐normalized data indicate more pronounced relative geometric remodeling in young mice when interpreted in relation to their smaller pre‐infarct ventricular volumes. Representative CMR images show the differences in ventricle volume over time (Figure 4D).

**FIGURE 4:**
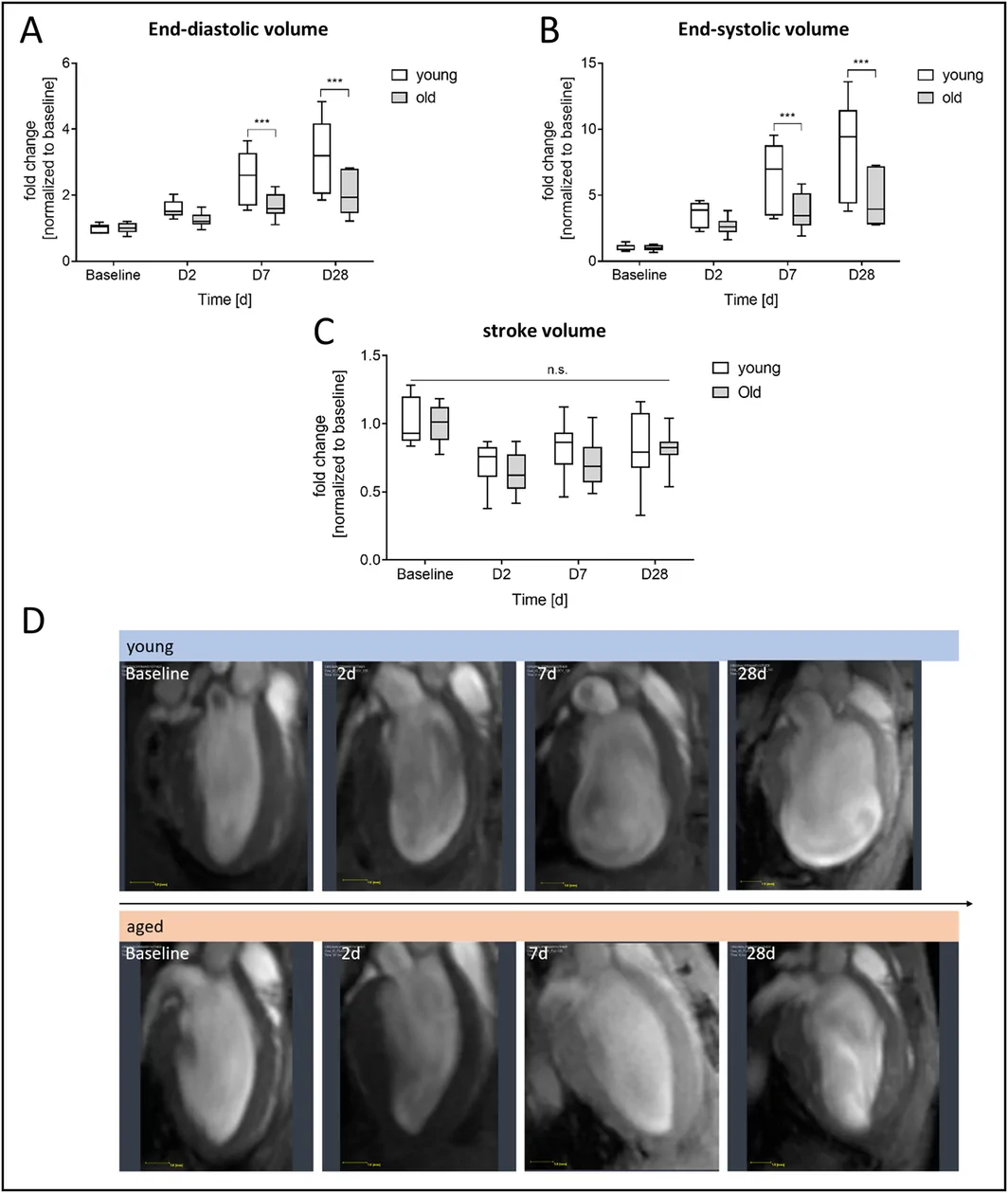
Changes of the ventricle volume following MI, normalized to baseline conditions. (A, B) End‐diastolic and end‐systolic volume increased after LAD‐ligation. Notably, comparison of both groups showed a more distinct incline of the volume in young animals. (C) Moreover, MI results in a reduction of the stroke volume that slightly recovers over time in both young and aged animals. (D) Representative CMR images of the long axis in young and aged animals at distinct time points, showing reduced ventricle volume after MI. Linear mixed‐effects model, three‐way ANOVA, followed by Sidak post hoc test, ***p < 0.001, *n* = 7–11.

In addition to chamber dilatation, MI‐induced remodeling was also associated with changes in myocardial mass. Diastolic mass increased in both age groups after infarction. In young mice, diastolic mass rose by approximately 1.5‐fold at Day 7 and showed a moderate decline by Day 28 (Figure 5A), whereas a comparable increase was observed in old mice (Figure 5B).

**FIGURE 5:**
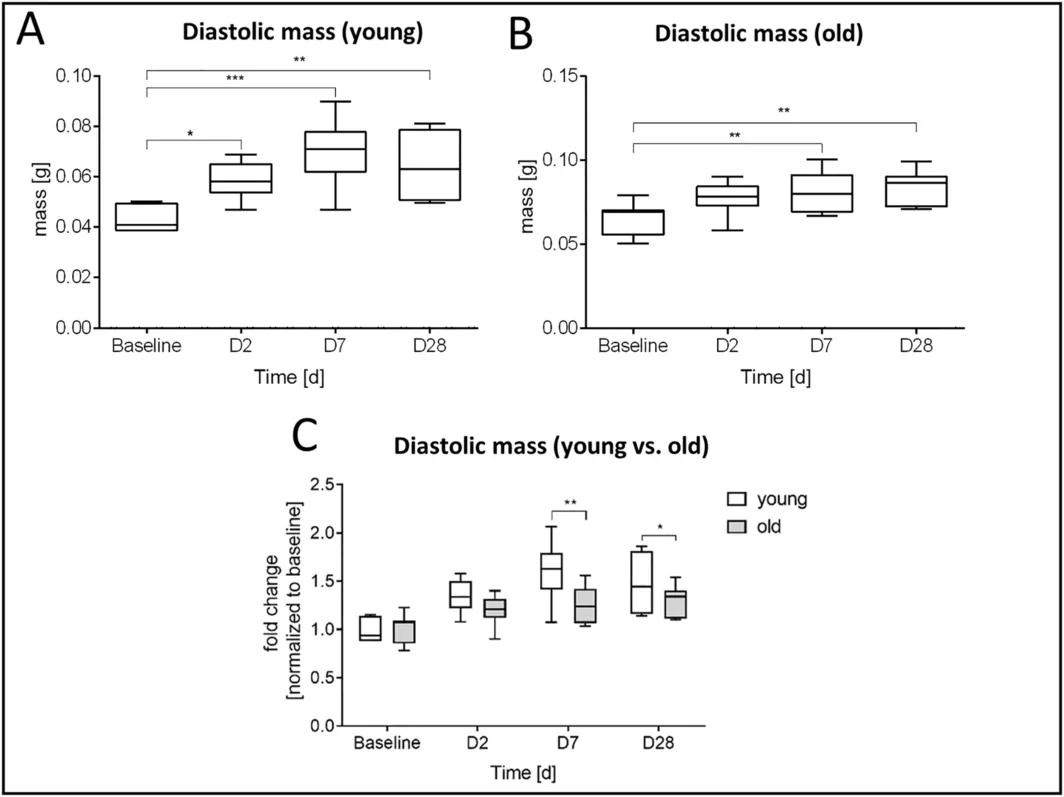
Effect of MI‐induced remodeling on heart tissue mass. (A, B) Both groups demonstrated an inclined diastolic mass after MI‐induction. (C) However, detected heart weight was significantly higher in old mice if compared to their younger counterparts. One‐way ANOVA, Sidak's post hoc test (A, B), Linear mixed‐effects model, three‐way ANOVA, followed by Sidak post hoc test, *n* = 7–11, *p < 0.05, **p < 0.005, ***p < 0.001.

Comparison of absolute diastolic mass values showed that old mice had significantly higher values overall across time points (*p* = 8.94e‐05) (Figure S1B). Diastolic mass also changed significantly over time (*p* = 2.73e‐08), but the age‐by‐time interaction was not significant (*p* = 0.6153), indicating no statistical evidence for different longitudinal trajectories between young and old mice. However, baseline‐adjusted analyses showed that baseline diastolic mass significantly predicted follow‐up values. After adjustment for baseline, old mice still exhibited significantly higher diastolic mass at Day 2 (*p* = 0.0451) and Day 28 (*p* = 0.0338), whereas the age effect was not significant on Day 7.

To assess proportional changes, baseline‐normalized diastolic mass values were calculated. Here, we found significant effects of age (*p* = 0.0231) and time (*p* = 1.38e‐08), whereas the age‐by‐time interaction did not reach statistical significance (*p* = 0.0910). Post hoc comparisons showed significant differences between young and old mice on Day 7 and Day 28 (Figure 5C), suggesting age‐related differences in relative diastolic mass remodeling at later stages of the healing process.

### 3.3 Ventricle Contractility Following Permanent LAD Occlusion

To quantify ventricular contractile function in greater detail, feature‐tracking analysis was performed, and strain data were acquired for GCS, GLS, GRS_Lax_, and GRS_Sax_. As expected, the deformation capacity of the healthy heart was higher in young animals as shown by the calculated baseline values for all strain variants (Figure 2).

Mixed‐effects analyses of the raw strain values demonstrated significant time effects for all strain parameters (*p* < 0.0001), reflecting marked post‐MI impairment of myocardial deformation. However, neither the main effect of age nor the age‐by‐time interaction was significant for GCS, GLS, GRS_SAX_, or GRS_LAX_, indicating no robust differences in absolute strain trajectories between young and old mice. In baseline‐adjusted mixed‐effects models restricted to post‐MI time points, GLS was the only strain parameter showing significant effects of time (*p* = 0.0325), baseline (*p* = 0.0454), and age‐by‐time interaction (*p* = 0.0363), suggesting a differential post‐MI GLS trajectory between young and old mice (Figure S2).

Following normalization to pre‐infarct baseline conditions, relative strain values showed a similar overall pattern, with significant time effects for all parameters (*p* < 0.0001) but no significant global age‐by‐time interactions. Nevertheless, post hoc comparisons revealed significant group differences on Day 7 for normalized GCS, GRS_LAX_ GRS_SAX_. Taken together, these findings indicate that MI‐induced a profound deterioration in strain parameters in both age groups, while baseline‐adjusted GLS emerged as the only strain measure providing statistical evidence for an age‐dependent difference in post‐MI remodeling (Figure 6).

**FIGURE 6:**
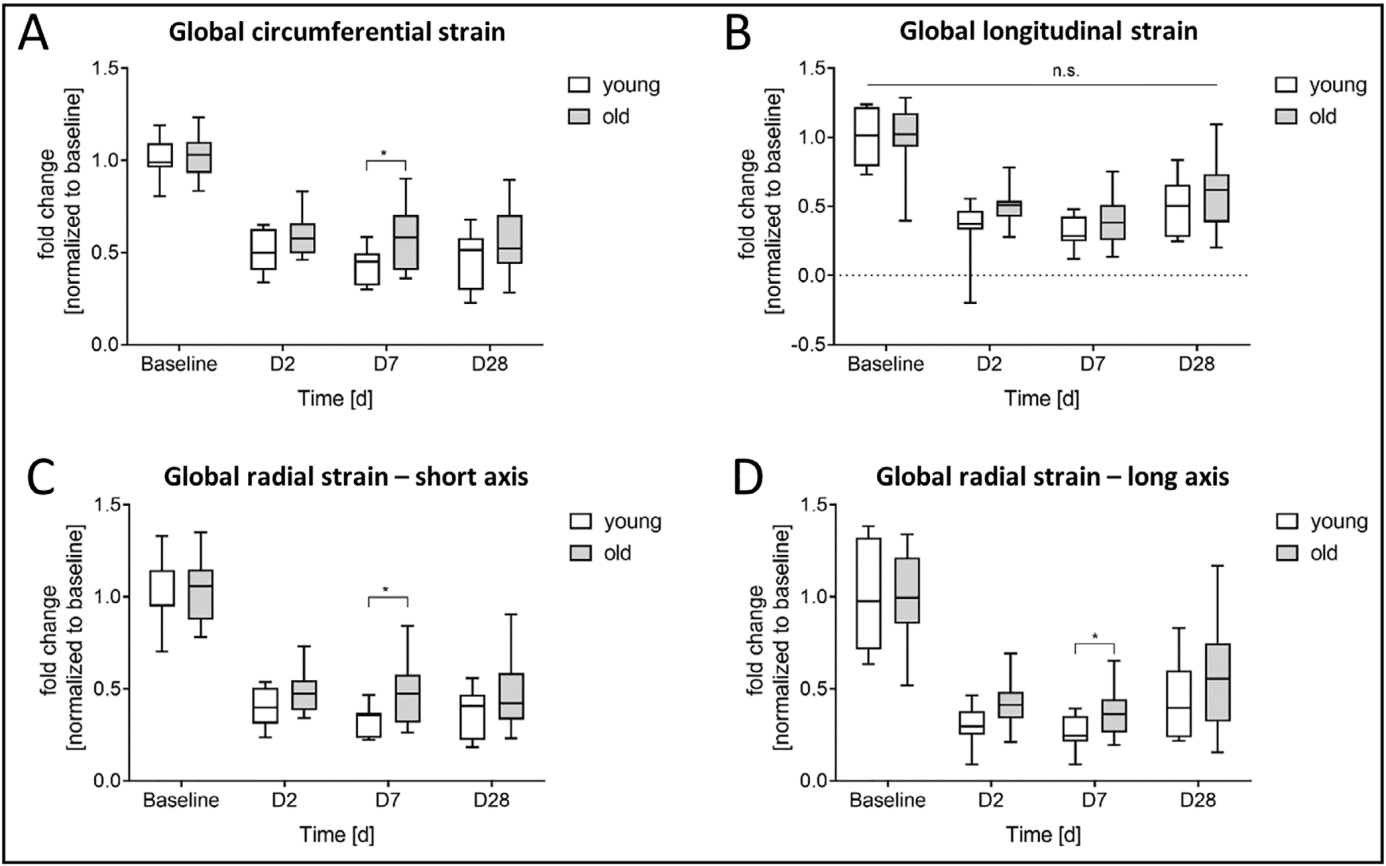
Measurement of heart contractility by strain analysis. To evaluate contraction capacity, feature tracking analysis was performed and (A) GCS, (B) GLS, (C) GRS_LAX_ and (D) GRS_SAX_ were calculated. Following MI, contraction capacity of the heart dramatically decreased in both groups for all detected strain variants. In young animals, MI results in lower contractility values if compared to pre‐infarct conditions. Linear mixed‐effects model, three‐way ANOVA, followed by Sidak post hoc test, *p < 0.05, *n* = 7–11.

### 3.4 Histological Analysis of Infarcted Hearts

Next, we asked whether the differences in morphology, contractility, and cardiac performance may also be reflected on the histological level. For this purpose, hearts were excised and scar size was evaluated in two different groups at Day 7 and Day 28 after MI. For visualization of the scar tissue, sections were stained with Masson Trichrome solution and labeled with antibodies targeting collagen V that have been recently reported to play a central role in the process of LV dilation in mice 1 week after MI (Yokota et al. 2020).

The age‐dependent comparison showed no statistically significant differences in fibrotic tissue on Day 7 and Day 28 (Figure 7A,B). However, a more distinct difference between both groups was found in hearts 7 days after LAD occlusion, showing less fibrotic areas in aged animals (D7: young: 0.61% ± 0.08% vs. old: 0.48% ± 0.08%, *p* = 0.927; D28: young: 0.43% ± 0.097% vs. old: 0.40% ± 0.08%, *p* > 0.99).

**FIGURE 7:**
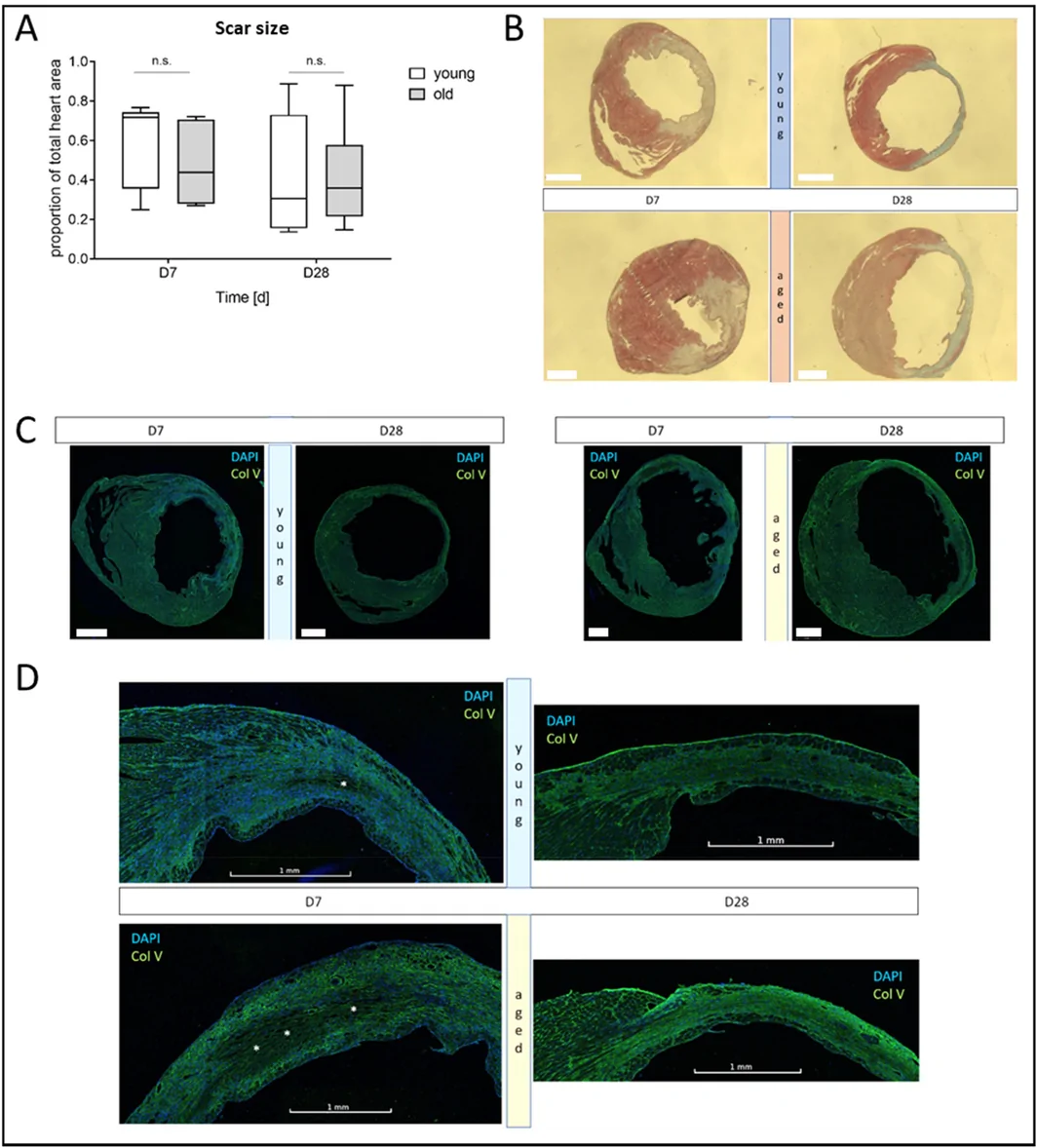
Analysis of scar tissue of infarcted hearts. (A) Quantitative evaluation showed a decline in scar tissue from D7 to D28, yet no differences between young and old animals were detected. Students *t*‐test, *n* = 6–10. (B) Representative images of heart slices labeled with Masson Trichrome and (C) heart slices labeled for Collagen V showed increased signal intensity at scar areas. (D) Higher magnification of the scar tissue revealed cell‐free areas at Day 7 post MI, predominantly in heart sections of aged animals. Scale bar 1 mm. Two‐way ANOVA, followed by Tukey post hoc test, *n* = 7–11.

After labelling tissue sections for collagen V and cell nuclei, we identified regions that were negative for both markers, suggesting the presence of cell‐free, necrotic areas, which demonstrated similar patterns of distribution in both remote myocardium and scar. At Day 7 these necrotic islets were present in both groups, but more limited in size and number in young mice, which might suggest faster healing in young animals (Figure 7C,D).

## 4 Discussion

Elderly patients have a higher risk for both to experience and dying from AMI (Shih et al. 2011). Clinical data from the era of treating MI with thrombolysis as the first line therapy for reperfusion demonstrated an age‐dependent increase in cardiac rupture rates (Maggioni et al. 1993). Furthermore, both clinical and preclinical studies have shown adverse effects of advanced age on left ventricular remodeling and cardiac function post‐MI (Bujak et al. 2008; Gould et al. 2002; St John Sutton et al. 1997). As a consequence, experts in the field cautioned more than a decade ago against the extrapolation of findings from young animal models to elderly human patients (Shih et al. 2011; Jugdutt and Jelani 2008). Yet, particularly in the field of cardiac regeneration, young mice of about 10 weeks of age still remain the most commonly used animal model (Lang et al. 2023).

### 4.1 Age‐Dependent Differences of Cardiac Function and Geometry

To address this gap, we first assessed the impact of age on cardiac function in healthy animals. Both size (EDV and ESV) and weight of the heart as well as stroke volume were higher in old mice. LVEF values were similar between both groups, whereas cardiac contractility, that is, circumferential, radial and longitudinal strain, were lower in old mice, yet not reaching a statistical significance.

Several research groups have analyzed age‐dependent changes of cardiac function and morphology in healthy mice. However, the preclinical studies described in the following have applied echocardiography and calculated both ventricular volumes and LVEF from the parasternal view which is inferior to the four‐chamber view using the Simpson method. For example, Chiao et al. reported slightly higher values for both end‐diastolic (LVEDD) and systolic dimension (LVESD) of the left ventricle in old (18–24 months) compared to young (6–9 months old) C57BL/6J mice without reaching a level of significance (Chiao et al. 2012). It is important to note that the authors used animals of both sexes, which hampers data interpretation due to the differences between male and female animals (see below).

In another study, the comparison of young (2–3 months) and old (24 months) C57BL/6J mice also showed higher LVEDD and LVESD in old mice, while FS was slightly decreased, yet not reaching the level of statistical significance. Mass of the left ventricle was significantly higher in old animals (Bujak et al. 2008). Similarly, longitudinal datasets from aging C57BL/6J mice (6–20 months) demonstrated significant morphological and functional changes of the heart during the aging process (de Lucia et al. 2019). LVEDD, LVESD, and LVM increased significantly, whereas FS and LVEF were found to be decreased in older animals. The authors also included deformation analyses and reported a declined global radial strain and an increase of global longitudinal strain. In contrast, no differences in LV dimensions and systolic function were reported by Derumeaux et al., who analyzed young (2–3 months) and old (6–18 months) male C57BL/6J mice. Yet, LV mass was significantly higher and strain rate significantly lower in old mice (Derumeaux et al. 2008).

The extent of age‐related changes on cardiac morphology and function is also influenced by sex as reported by Koch and colleagues. Both male and female FVB/N mice were monitored from the age of 3 months up to 16 months (Koch et al. 2013). Mal mice exhibited a significantly increase of EDV, ES and LVM, while no changes were detected in female animals. On the contrary, LVEF increases slightly in females and decreases significantly in male mice. Likewise, circumferential strain remains similar over time in females but shows a trend of progressive decline in males.

No profound differences on cardiac geometry and contractility between sexes were reported by Zhang et al. who compared young (2 months) and old (24 months) mice of both sexes, yet not mentioning the mouse strain (Zhang et al. 2021). Aging tended to lower GLS, GCS, and GRS values, yet only reaching a level of significance in males for GLS and GCS in females. Moreover, they found significantly higher EDV, ESV, SV, and LVM in both sexes and no difference in LVEF.

Since there is a profound impact of sex and mouse strain on cardiac aging, it is important to consider these parameters in the study design and when comparing imaging data across different studies. The distinct, often contradictory, observations of LV geometry and contractility in previous publications may be explained by the limitations of echocardiography used for cardiac imaging in mice. Hence, the application of small animal dedicated CMR represents a more precise tool to overcome this challenge.

### 4.2 Differences of LV Remodeling Heart Between Old and Young

We performed the first CMR based study to compare progressive LV remodeling in young and old mice by a longitudinal protocol starting before MI and followed by CMR scans 2, 7 and 28 days post‐MI. Surprisingly, we observed a more dramatic relative LV dilatation in young animals despite no differences in scar size at Day 7 and Day 28. LVEF and SV were comparable between both groups, on both the absolute and proportional level. The data also indicate that young and old mice may reach similar absolute post‐MI values despite differing in the proportional extent of change from baseline. Based on these novel findings, we had to reject our first hypothesis that MI induces a more severe LV remodeling and impaired systolic function in aged mice. Furthermore, immunohistochemistry analysis revealed no difference in collagen Type V distribution within the scar tissue, failing to support our second hypothesis of a reduced collagen V deposition in old animals.

Gould et al. (2002) have already questioned the use of young mice as an appropriate model for post‐MI heart failure more than 20 years ago. They induced AMI by permanent ligation of the LAD in young (2 months) and older (14 months) male C57BL/6N mice and detected significantly larger infarcts in young mice after 24 h, but more extensive scar expansion in the aged group.

A subsequent groundbreaking study was published in 2008 by Bujak et al. (2008) who compared myocardial healing and left ventricular remodeling after transient occlusion of the LAD for 60 min in young (2–3 months) and old (24 months) C57BL/6J mice. Echocardiographic assessment at Day 7 revealed significantly larger LVEDD and LVESD with significantly lower fractional shortening (FS) in old animals. Histological data exhibited delayed neutrophil and macrophage infiltration and impaired phagocytosis of dead cardiomyocytes after 1 and 3 days in aged mice, accompanied by less collagen deposition in the scar at Day 7. Yabluchanskiy et al. (2015) examined LV remodeling after permanent LAD occlusion in C57BL/6J mice aged from 11 to 36 months of both sexes. Echocardiography at Day 7 revealed a progressive increase in left ventricular dilation with age, while scar size assessed by histological analysis showed no difference.

Only one study used CMR to compare post‐MI remodeling in mice (Walaszczyk et al. 2019). Here, the authors describe a more pronounced decline of LVEF, higher LV mass, and larger cardiomyocytes in aged mice. However, crucial methodological parameters, like sex, mouse strain, type of infarct, exact age, and imaging details were not specified, thus limiting data interpretation.

When considering global LV dimension and LVEF of the limited number of mouse studies, evidence appears relatively consistent: MI healing is impaired in aging mice resulting in a more pronounced LV dilation. Unexpectedly, we found that progressive LV dilation was significantly more pronounced in young female C57BL/6 mice. However, a closer look into these few existing studies reveals a far less coherent picture: All of these reports used C57BL/6 mice. Two studies used only male mice (Gould et al. 2002; Bujak et al. 2008), one study included both sexes (Yabluchanskiy et al. 2015) and one did not even report the gender of the animals used (Walaszczyk et al. 2019). Methodological differences further complicate interpretation: Two studies applied permanent ligation (Yabluchanskiy et al. 2015; Gould et al. 2002) and one study transient ligation to induce myocardial infarction (Bujak et al. 2008), while one did not provide any details concerning infarct induction (Walaszczyk et al. 2019). Furthermore, imaging modalities also varied as two studies used echocardiography (Bujak et al. 2008; Yabluchanskiy et al. 2015) for in vivo imaging and one used CMR (Walaszczyk et al. 2019). Three of the studies which provide details on the anesthetic during imaging used a mixture of ketamine and xylazine (Bujak et al. 2008; Yabluchanskiy et al. 2015; Gould et al. 2002).

These methodological details differ from our study design, in which we used exclusively female mice, isoflurane for anesthesia and a well‐established CMR‐based imaging protocol. Therefore, differences between published dataset may be explained by the critical impact of gender (Jain et al. 2002), infarct model (Lang et al. 2021; Raitza et al. 2025), anesthesia (Lindsey et al. 2018) and the high inter‐observer variability of echocardiography in mice. In addition, M‐mode imaging is not recommended for LV assessment of post‐MI due to the non‐uniform remodeling of the ventricle that includes thinning of the infarct area and thickening of the remote myocardium and may account for discrepancies (Lindsey et al. 2018).

Despite the divergence from previous animal studies, our findings are supported by a recent clinical report from Guo and co‐workers. The authors compared young and old STEMI patients within the first week of admission and after a follow‐up period of 3 months based on cardiac CMR. Despite similar infarct characteristics, younger patients were more prone to adverse remodeling compared to older ones (Guo et al. 2023). These detected differences in LV remodeling were not reflected on the histopathological level. Analysis of scar size showed no age‐dependent variations, which is consistent with former reports (Deten et al. 2005; Yabluchanskiy et al. 2015).

Interestingly, we identified cell‐free areas within infarcted heart sections, termed necrotic islands, in both groups. Notably, young mice exhibited a reduced number and size of these cell‐free regions, suggesting more rapid structural healing of the myocardial defect. Hence, one may speculate that an accelerated healing process does not necessarily translate into improved cardiac remodeling. Yet, this hypothesis has to be addressed in more detail in future studies.

### 4.3 Limitations of the Study

As discussed above, several studies reported sex‐dependent differences in cardiac function and post‐MI remodeling. In this context, it is important to mention that the present study was conducted exclusively in female C57BL/6 mice. The use of a single sex was intended to reduce biological heterogeneity and to enable a more controlled longitudinal comparison within a uniform experimental setting. However, since several earlier preclinical studies on age‐dependent post‐MI remodeling were conducted in male animals, direct comparison across studies should be made with caution (Bujak et al. 2008; Gould et al. 2002). Sex‐related differences in cardiac aging and post‐infarction remodeling may contribute to divergent findings between experimental studies.

When interpreting our longitudinal CMR data, it should also be considered that only animals surviving surgery and follow‐up period are included in the serial analyses. Therefore, a certain degree of survival‐related selection bias cannot be fully excluded. However, as post‐operative mortality and exclusion rates were comparable between young and old mice, a major age‐dependent selection is unlikely (Figure S3).

For MI induction we have used permanent LAD ligation rather than an ischemia/reperfusion model. This allowed us to generate a robust and standardized severe ischemic insult and enabled us to assess the age‐dependent ventricular remodeling by serial CMR without the additional biological variability introduced by reperfusion and myocardial salvage. However, this study design does not capture the reperfusion component that characterizes current standard care in many patients with acute myocardial infarction, including potential age‐dependent differences in susceptibility to reperfusion‐associated oxidative injury (An et al. 2026). Future studies using ischemia/reperfusion models will be required to determine whether the age effect on post‐MI remodeling differs when reperfusion injury is superimposed on the initial ischemic event.

A further limitation of the study is that we did not directly quantify the acute ischemic area or infarct size at the time of surgery. Although LAD ligation was performed in a standardized manner by an experienced surgeon and successful ligation was confirmed intraoperatively by blanching of the target region, we cannot exclude that ligation at the same anatomical level may have affected a different proportion of the LV in smaller versus larger hearts. Early post‐MI LVEF at Day 2 was used as an indirect functional surrogate to confirm successful infarction and exclude animals with insufficient functional deterioration. Therefore, potential group differences in the relative size of the initial ischemic insult should be considered when interpreting subsequent remodeling patterns.

## 5 Conclusion

In summary, this is the first mouse study to provide longitudinal CMR imaging to characterize differences in LV remodeling after permanent ligation in female C57BL6 mice. We demonstrated that LAD occlusion leads to a significantly pronounced LV dilatation and a more reduced myocardial contractility in young mice when accounting for the markedly different pre‐infarction cardiac geometry of young and old animals. From a biological perspective, comparable absolute changes may represent different remodeling burdens depending on the starting condition of the ventricle. However, additional studies will be required to fully elucidate the mechanisms underlying cardiac remodeling. In this context, the dedicated assessment of diastolic function and relaxation peaks would be of particular interest, especially given the higher diastolic mass observed in old animals.

Nevertheless, our findings challenge the hypothesis of an age‐dependent increase of LV remodeling after MI in female C57BL/6 mice.

## Author Contributions

Conception and design of the study: C.I.L., R.D., B.J.K. Acquisition of data: H.L., P.W., A.D., R.G., P.V., T.L. Analysis and interpretation of data: H.L., P.W., A.D., M.R., M.S., F.G.M., R.D. Drafting or revising the manuscript: H.L., P.W., A.D., M.S., M.R., T.L., H.I., F.G.M., B.J.K., B.V., R.D., C.I.L. All authors have approved the final article.

## Funding

This work was supported by the Federal Ministry of Education and Research Germany (FKZ 0312138 A and FKZ 316159), the State Mecklenburg‐Western Pomerania with EU Social Funds (ESF/IVWM‐ B34‐0030/10 and ESF/IVBM‐B35‐0010/12), the DFG (DA1296/6 – 1, VA1797/1–1), and the German Heart Foundation (F/01/12). In addition, the project was supported by the EU Social Fund (ESF/14‐ BM‐A55‐0024/18 and ESF/14‐BM‐A55‐0027/18) and the BMBF (VIP+00240). CIL was supported by the Clinician Scientist Program of Rostock University Medical Center and the German Heart Research Foundation (F/34/15). MS was supported with a Kaltenbach scholarship by the German Heart Foundation (K/40/19).

## Ethics Statement

This study was conducted with the approval of the federal animal care committee of the Landesamt für Landwirtschaft, Lebensmittelsicherheit und Fischerei Mecklenburg‐Vorpommern (LALLF) in Germany (reference number LALLF M‐V 7221.3‐1‐ 032/20).

## Conflicts of Interest

The authors declare no conflicts of interest.

## Supporting information


**Data S1:** acel70748‐sup‐0001‐DataS1.docx.

**Figure S1:** Effect of MI on LVEF and diastolic mass in young and old mice. Linear mixed‐effects model, three‐way ANOVA, followed by Sidak post hoc test, *n* = 7–11.
**Figure S2:** Measurement of heart contractility by strain analysis. To evaluate contraction capacity feature tracking analysis was performed and GCS, GLS, GRSLAX, and GRSSAX were calculated. Linear mixed‐effects model, three‐way ANOVA, followed by Sidak post hoc test, *n* = 7–11.
**Figure S3:** Contingency plot of included, dead and excluded animals used in the CMR and histology cohort.

## Data Availability

The datasets generated during and/or analyzed during the current study are available from the corresponding author on reasonable request.
