# Supplementary material for: Young Mice Exhibit Increased Relative Ventricular Remodeling After AMI—A Longitudinal CMR Study
Source: Aging Cell. 2026 Sep 30;25(10):e70748. doi: 10.1111/acel.70748 (PMC13627973; doi:10.1111/acel.70748)
Supplement: Supplementary file 2 [file ACEL-25-e70748-s002.docx]

Figure Captions, supplemental figures

**Fig S1: Effect of MI on LVEF and diastolic mass in young and old mice.** Linear mixed-effects model, three-way ANOVA, followed by Sidak post hoc test, n=7-11.

**Fig S2: Measurement of heart contractility by strain analysis.** To evaluate contraction capacity feature tracking analysis was performed and GCS,GLS, GRSLAX and GRSSAX were calculated. Linear mixed-effects model, three-way ANOVA, followed by Sidak post hoc test, n=7-11.

**Fig S3: Contingency plot of included, dead and excluded animals used in the CMR and histology cohort**
